# Host CD3^+^ T-cells can significantly modulate phage treatment effects on bacterial bioburden in mouse models

**DOI:** 10.3389/fmicb.2023.1240176

**Published:** 2023-09-11

**Authors:** Renhua Li, Michael Rouse, Brendon T. Pace, Scott F. Grey, Kimberly Mclaughlin, Seth A. Schobel, Mark P. Simons

**Affiliations:** ^1^Uniformed Services University of the Health Sciences, Bethesda, MD, United States; ^2^Surgical Critical Care Initiative (SC2i), Uniformed Services University (USU), Bethesda, MD, United States; ^3^Henry M. Jackson Foundation for the Advancement of Military Medicine, Inc, Bethesda, MD, United States; ^4^Walter Reed National Military Medical Center, Bethesda, MD, United States; ^5^Eastern Virginia Medical School, Norfolk, VA, United States; ^6^Naval Medical Research Center, Silver Spring, MD, United States

**Keywords:** wound healing, phage treatment, bacterial bioburden, CD3^+^ T-cells, systems biology, bi-directional relationship between bacterial burden and host immune responses

## Abstract

Wound healing is a complex system including such key players as host, microbe, and treatments. However, little is known about their dynamic interactions. Here we explored the interplay between: (1) bacterial bioburden and host immune responses, (2) bacterial bioburden and wound size, and (3) treatments and wound size, using murine models and various treatment modalities: Phosphate buffer saline (PBS or vehicle, negative control), doxycycline, and two doses of *A. baumannii* phage mixtures. We uncovered that the interplay between bacterial bioburden and host immune system may be bidirectional, and that there is an interaction between host CD3^+^ T-cells and phage dosage, which significantly impacts bacterial bioburden. Furthermore, the bacterial bioburden and wound size association is significantly modulated by the host CD3^+^ T-cells. When the host CD3^+^ T-cells (x on log10 scale) are in the appropriate range (1.35 < x < = 1.5), we observed a strong association between colony forming units (CFU) and wound size, indicating a hallmark of wound healing. On the basis of the findings and our previous work, we proposed an integrated parallel systems biology model.

## 1. Introduction

Wound healing is impacted by a complex system involving treatments, microbial bioburden, and both cellular and molecular responses of the host. Previous studies focused on single or pairwise aspects of the triangle, making it challenging to get a full, multi-dimensional picture of the wound healing system. Furthermore, “time” provides yet another dimension of variation and dependence to the system. The ultimate outcomes of the system related to wound care are either successful closure or dehiscence of the wound.

In the wound healing system, the host immune system plays significant roles in response to microbial infections and in regulation of wound healing (Krafts, 2010; Cooke, 2019). Regarding innate immune responses, circulating cell types, such as neutrophils and M1 macrophages, are among the key players that can release various inflammatory cytokines, such as tumor necrosis factor alpha (TNF-a), interleukin (IL)-1b, IL-6, IL-8, and chemokines, to trigger inflammatory responses (Krafts, 2010; Julier et al., 2017). On the other hand, after successful elimination of pathogens, the immune system can switch to an immunosuppressive state (Cooke, 2019). For example, cell types, such as N2 neutrophils and M2 macrophages, produce anti-inflammatory cytokines and growth factors that promote the generation and expansion of immunosuppressive cells, including NK regulatory cells (NKregs) and T regulatory cells (Tregs). Furthermore, M2 macrophages have demonstrated that they are one of the essential factors for scaffolding, re-epithelialization, and ultimately, successful wound healing (Snyder et al., 2016). This is the hallmark of transition from inflammation to anti-inflammation, sparking the initiation of the natural wound healing process (Krafts, 2010; Julier et al., 2017).

Various T-cell types play special roles in wound healing. In a full-thickness skin excisional wound study in Wistar rats, for example, CD3^+^ T lymphocytes in the wound bed were found to associate primarily with the phase of granulation tissue formation to promote wound healing (Agaiby and Dyson, 1999). Similar findings of CD3^+^ T-cells promoting and increasing the rate of wound healing were also reported during *in vitro* studies using human skin biopsies (Toulon et al., 2009). In skin wounds, the presence and migration of γσ + T lymphocytes are important for hemostasis, inflammation, proliferation and remodeling (Hu et al., 2022). γσ + T cell receptor-bearing dendritic epithelial T-cells (DETCs) are specialized T-cells that reside in the epithelial layer of human and murine skin. They recognize skin damage using T-cell receptors that are activated by antigens expressed by stressed keratinocytes (Jameson et al., 2002; Alexander-Savino et al., 2016). The removal of γσ + DETCs from murine skin significantly impaired keratinocyte proliferation in damaged skin, thus increasing wound healing time and demonstrating that these T-cells are important for the healing process (Hu et al., 2022). While other T-cell types are associated with enhanced wound healing, numerous studies have shown that the activity of CD8^+^ cytotoxic T cells in skin wounds can slow down the healing process (Krafts, 2010; Cooke, 2019). However, another study on the role of CD4^+^ and CD8^+^ T-cells in mouse skin wound healing found that the absence of either CD4 or CD8 lymphocytes changes infiltration of inflammatory cells and profiles of cytokine expression, but does not impair healing (Chen et al., 2014). In response to burn injuries, Labuz et al. (2023) found that conventional CD3^+^ T cells and MAIT cells produce elevated levels of critical pro-inflammatory cytokines than in non-burn tissue.

As antibiotic resistant strains of bacteria become more prevalent, the development of alternative sustainable treatments, such as bacteriophage (phage), is key to either complement or augment antimicrobial drugs (Lin et al., 2017). Compared to broad-spectrum antibiotics, phage therapy has the advantage of being highly specific toward target bacterial pathogens (Loc-Carrillo and Abedon, 2011; Sarker et al., 2017). Studies on murine models indicated that neutrophil-phage synergy is essential for infection clearance and the resolution of pneumonia (Roach et al., 2017). This “immunophage synergy” highlights the dynamic relationship between microbe and host immune system, and the impact a therapeutic agent can have on this interaction.

In the present study, using an established mouse wound infection model and multiple treatment modalities, we initiated to explore and represent the full picture about wound healing. We uncovered that the interplay between bacterial burden (CFU) and host immune responses is bidirectional. Similar to previous studies (Desgeorges et al., 2019), the host responses to CFU have a dynamic phase change from pro-inflammation to anti-inflammation. On the other hand, phage treatment delivered at 5 × 10^8^ total plaque forming units or PFU (phage-High) can significantly reduce CFU, compared to phage-Med (5 × 10^7^ total PFU), when there is a high percentage of CD3^+^ T-cells in the host immune cell population. Furthermore, we identified that when the T-cells (x on log10 scale) are in the appropriate range (1.35 < x < = 1.5) in mice, there is a strong correlation between CFU and wound size, indicating a signal for wound healing. These new findings clearly signify the importance of CD3^+^ T-cells – phage dosage synergy in the wound healing system, thus laying the foundation for future exploration of phage treatment in wound healing in humans.

## 2. Materials and methods

### 2.1. Mouse models and experimental design

**Figure 1:**
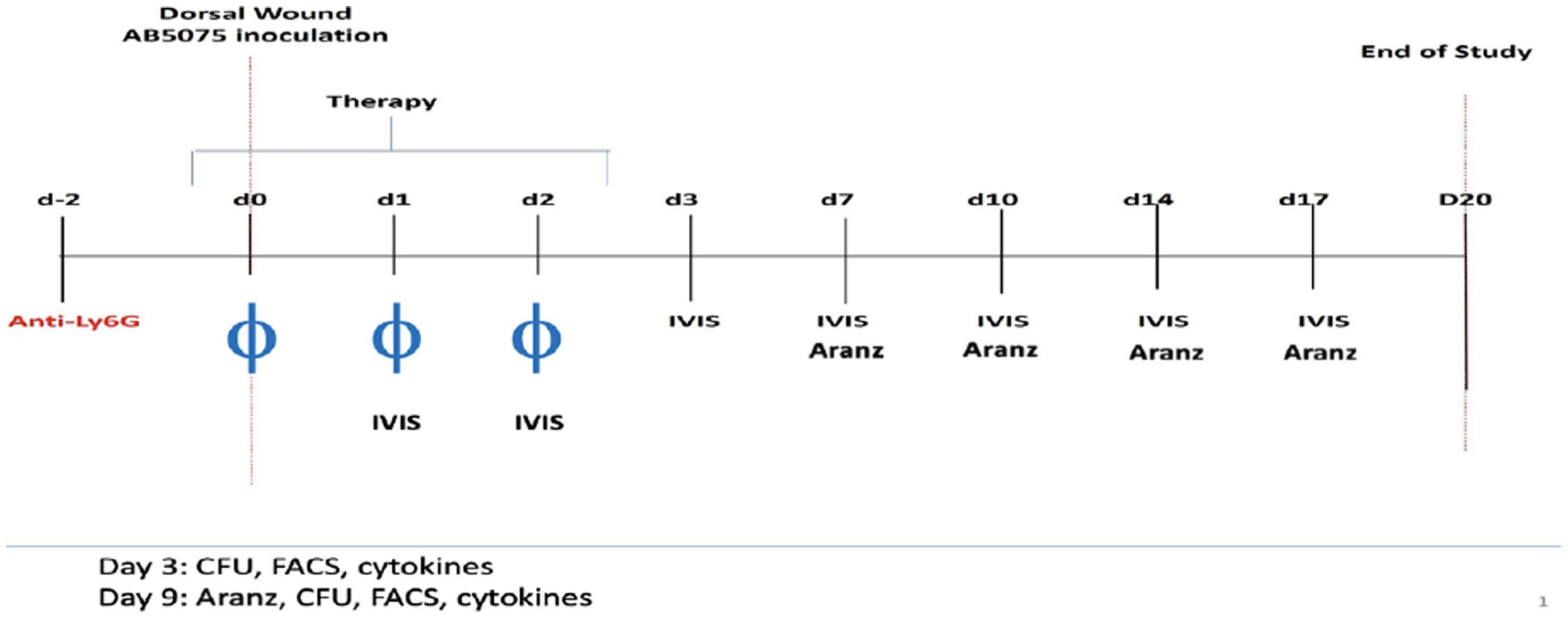
Schematic time course for the experiments. Two days before inoculation for transient immunosuppression, 6-week-old female BALB/c mice (*N* = 65) were administered 50 μg of recombinant anti-mouse Ly6G monoclonal antibodies intraperitoneally. On Day 0, full-thickness dorsal wounds were induced on the backs of mice which were inoculated with ∼5 × 10^6^ total colony forming units (CFU) of *A. baumannii* (strain AB5075:lux), followed by a Tegaderm bandage and sealed with Vetbond™ (3 M, St. Paul, MN). Wounded and infected mice were then randomly separated into four treatment groups: (1) phosphate-buffered saline (Vehicle, negative control), (2) doxycycline hyclate (Doxy), (3) phage 5 × 10^7^ total PFU (phage-Med), and (4) phage 5 × 10^8^ total PFU (phage-High). Doxycycline treatment was administered as a single topical 50 μl of 50 g regimen 2-h post-inoculation on Day 0. Vehicle and phage treatments were administered as topical regimens at 2-, 24-, and 48-h post-inoculation. *In vivo* imaging system (IVIS) was performed on mice to visualize and semi-quantify the bacterial burden at the indicated time points. The Aranz Silhouette system wound measurement device was used to image and measure the total wound area of mice longitudinally over multiple days. We focused on comparing the measurements on Days 3 and 9, respectively.

Using an established mouse wound infection model, we aimed to understand the dynamic wound healing system. The experimental design is shown schematically in Figure 1. Six-week-old female BALB/c mice (*N* = 65) were administered intraperitoneally 50 μg of recombinant anti-mouse Ly6G monoclonal antibodies 2 days before inoculation for transient immunosuppression (Rouse et al., 2020). On Day 0, full-thickness dorsal wounds were created on the mice which were inoculated with ∼5 × 10^6^ total colony forming units (CFU) of *A. baumannii* (strain AB5075:lux), followed by a Tegaderm bandage and sealed with Vetbond™ (3 M, St. Paul, MN). Wounded and infected mice were then randomly separated into four treatment groups: (1) phosphate-buffered saline (PBS) vehicle control (Vehicle), (2) doxycycline hyclate (Doxy), (3) phage 5 × 10^7^ total PFU (phage-Med), and (4) phage 5 × 10^8^ total PFU (phage-High) (Rouse et al., 2020). Doxycycline treatment was administered as a single topical 50 μg regimen 2-h post-inoculation on Day 0. Vehicle and phage treatments were administered as a topical solution between the layers of Tegaderm and wound at 2-, 24-, and 48-h post-inoculation. The five member phage mixture (AbArmy ϕ1, AbNavy ϕ1, AbNavy ϕ2, AbNavy ϕ3, and AbNavy ϕ4) that was designed to be lytic against AB5075:lux was added at equal volumes to generate a stock concentration of 1 × 10^9^ PFU/mL and was described previously by Regeimbal et al. (2016). Mice were housed singly from Day 0 (inoculation) through Day 6. On Day 7 post-inoculation, each Tegaderm dressing was removed, and the wounds were left exposed to air for the remainder of the experiment. *In vivo* imaging system (IVIS) was performed on mice to visualize and semi-quantify the bacterial burden at the indicated time points. The Aranz Silhouette system wound measurement device was used to image and measure the total wound area of mice on Days 0 (baseline), 7, 9, and 11. Randomly selected mice were euthanized on Day 3 and 9 post-inoculation to excise wound beds for homogenization and microbial bioburden enumeration via serial dilution plating. Spleens were also excised, homogenized, processed for immunostaining, and analyzed using flow cytometry for host immune profiling (markers for T-cells, Neutrophils, Dendritic cells, and Macrophages).

Serum was collected at various time points and stored at –20°C to later assess systemic cytokine/chemokine levels. A custom Millipore 25-plex Mouse Cytokine/Chemokine Magnetic Bead Panel kit (MCYTOMAG-70 K-PMX, Burlington, MA, USA) was performed on the serum samples according to the manufacturer’s instructions to analyze the following cytokines/chemokines on the Luminex platform: G-CSF, IFN-γ, IL-1α and IL-1β, IL-2, IL-4, IL-5, IL-6, IL-7, IL-9, IL-10, IL-12p40, IL-12p70, IL-13, IL-15, IL-17, IP-10, KC, MCP-1 MIP-1a, MIP-1b, MIP-2, RANTES, TNFα, and GM-CSF. The total numbers of inbred mice used was 65 on day 3 and 9, respectively, but some measurements, such as Luminex assay, was conducted on a subset of the mice (*n* = 32) on Day3.

### 2.2. Statistical analysis methods

To determine how critical variables, such as treatments (TRT), time course (TIME), and various immune cell types, impact bacterial bioburden (CFU), we fit a multiple mixed effects model for CFU prediction. In the model, cell types or ratios of cell types (such as CD4:CD3 and CD8:CD3) are treated as random samples from the total immune cell population of the host. In contrast, TRT and TIME are treated as fixed effects, as are the interactions of TRT and the cell types (or the ratios of cell types). The sample size (*n* = 65) provided enough power to estimate these effects accurately. To avoid model overfitting and power reduction, the cytokine/chemokine biomarker data was excluded from the modeling, due to the limited sample size (n = 32) shared between Day 3 and 9. However, the cytokine/chemokine profiles at the two primary time points (Day 3 and 9) allowed us to investigate dynamic changes of the host immune responses after bacterial infections. Furthermore, we scrutinized the dynamic association between treatments and wound size, given the condition of bacterial bioburden; and between bacterial burden and host wound size, given the host immunological status. The missing values in wound size were accurately imputed by using the random forest based non-parametric method (Stekhoven and Buhlmann, 2012), based on such covariates as bacterial burden and host immune cell compositions (CD3, CD4:CD3, and CD8:CD3 ratios).

### 2.3. Ethics statement

The experiments were conducted according to the compliance policy set by the Committee of Laboratory Animal Resources and Institutional Animal Care and Use Committee (IACUC) at the Naval Medical Research Center. The study was conducted in accordance with the local legislation and institutional requirements.

### 2.4. Resource availability

The inbred mouse strain BALB/c was purchased from the Charles River Laboratories International, Inc. Both the data and R scripts are archived at the shared Google drive within the Surgical Critical Care initiative (SC2i), which are available upon request to the manager Dr. Scott F. Grey (scott.grey.ctr@usuhs.edu).

## 3. Results

### 3.1. Phase changes of the host immune system in response to dynamic alternations of bacterial bioburden

**Figure 2:**
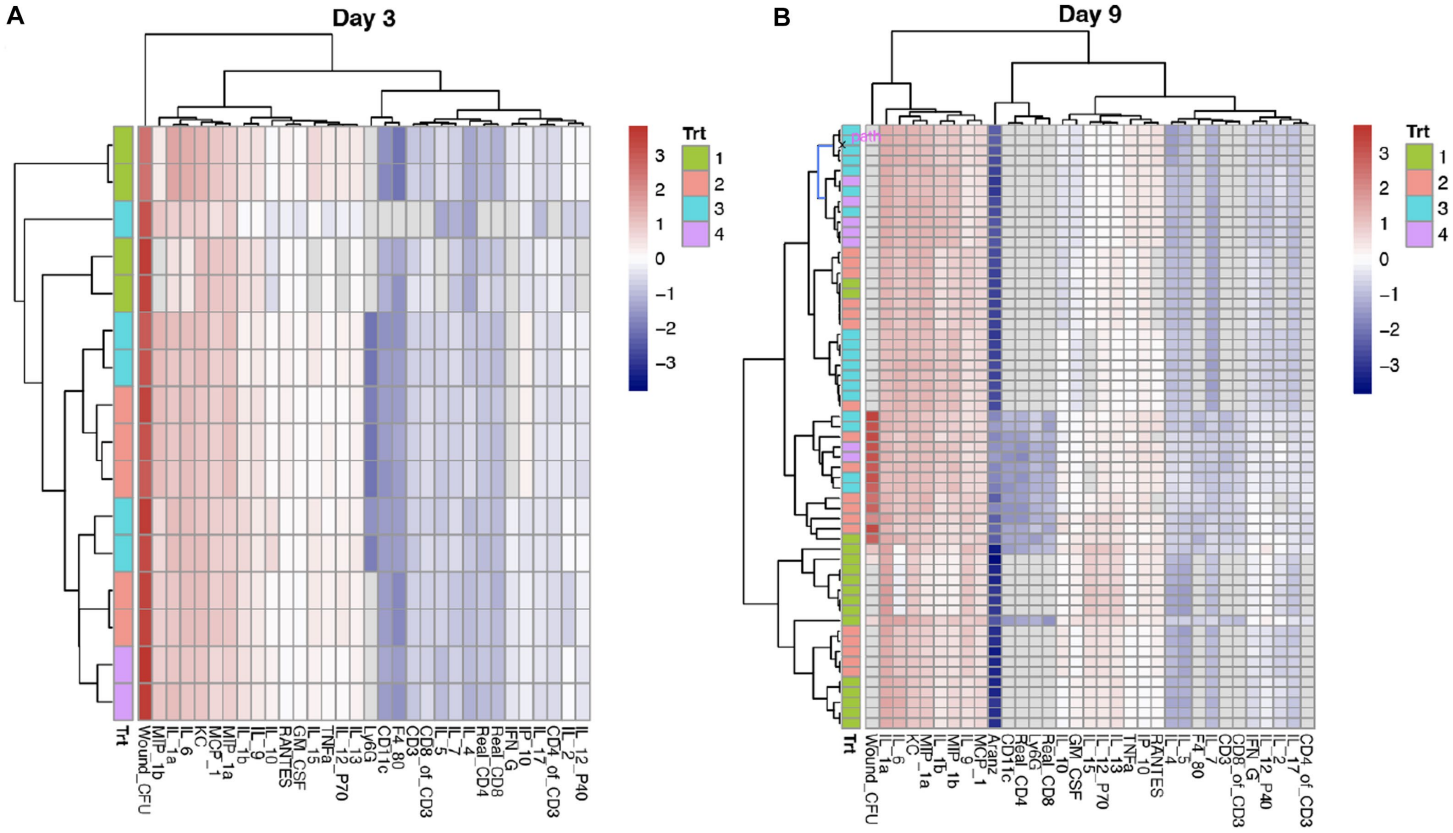
Comparison of cytokines and chemokines profiling on Days 3 and 9. **(A,B)** Each row represents a sample or mouse individual, while each column is a cytokine/chemokine biomarker or an immune cell type. Wound CFU is the measure of total colony forming units (CFU) of *A. baumannii* (strain AB5075:lux) at the wound site. Trt indicates the four treatments: 1 = Vehicle (control); 2 = phage-Med; 3 = phage-High; and 4 = doxycycline. After scaling across the samples at each time point, the heatmap shows the relative intensity of a value (*z*-score) within the matrix. The values that are highest are given a “red” color and the numbers that are lowest are given a “blue” color. While it may be challenging to separate the colors, the clustering is the gold standard to separate different groups of cytokines and coupled immune cell types. The cytokines and cell types in the same cluster indicate that they are closely related to one another. We observed that the red and blue colors are representative of respective pro- and anti-inflammatory biomarkers and coupled cell types. Compared to Day 3, Day 9 has a heatmap that has a significantly shrunk “Red” cluster and an expanded “Blue” cluster, indicating reducing of pro-inflammatory and increasing of anti-inflammatory.

We observed significant time-dependent changes in the cytokine/chemokine profiles, as shown by the two heatmaps of Day 3 (acute) and 9 (peak) in relation to course of infection, respectively (Figure 2). At each of the time points, there existed two distinct clusters (“Red” and “Blue”), corresponding to respective pro- and anti-inflammatory biomarkers that are coupled with immune cell types. On Day 3, the “Blue” cluster is composed of three subgroups: (1) the “Dark Blue” (or B1) that includes only three immune cell types: Ly6G (neutrophils), CD11c (dendritic cells), and F4-80 (or macrophages); (2) the “Light Blue” (or B2) that represents anti-inflammatory biomarkers (such as IL-2, IP-10, and IFNℽ) and ratio of CD4:CD3; and (3) the “Intermediate Blue” (or B3) that harbors anti-inflammatory biomarkers (such as IL-4 and IL-5) and coupled T-cells (Figure 2). In contrast, the “Red” cluster has only two subgroups: (1) the “Dark Red” (or R1) that includes proinflammatory biomarkers (such as IL-1a and IL-6) and cell types (such as killer cells or KC); and (2) the “Light Red” (or R2) that has proinflammatory biomarkers (such as IL-10 and TNFα) (Figure 2). Compared to Day 3, the Day 9 heatmap has a significantly reduced “Red” cluster and an expanded “Blue” cluster, due to the switching of R2 to the “Blue” cluster. These results clearly indicated a phase change of the host immune system in response to the dynamic alternation of bacterial bioburden (CFU). We then investigated how the phase change modulated the bacterial bioburden abundance and the dynamic association of bacterial bioburden and wound size.

### 3.2. Bacterial bioburden is impacted by multiple factors including host immunological status

**Figure 3:**
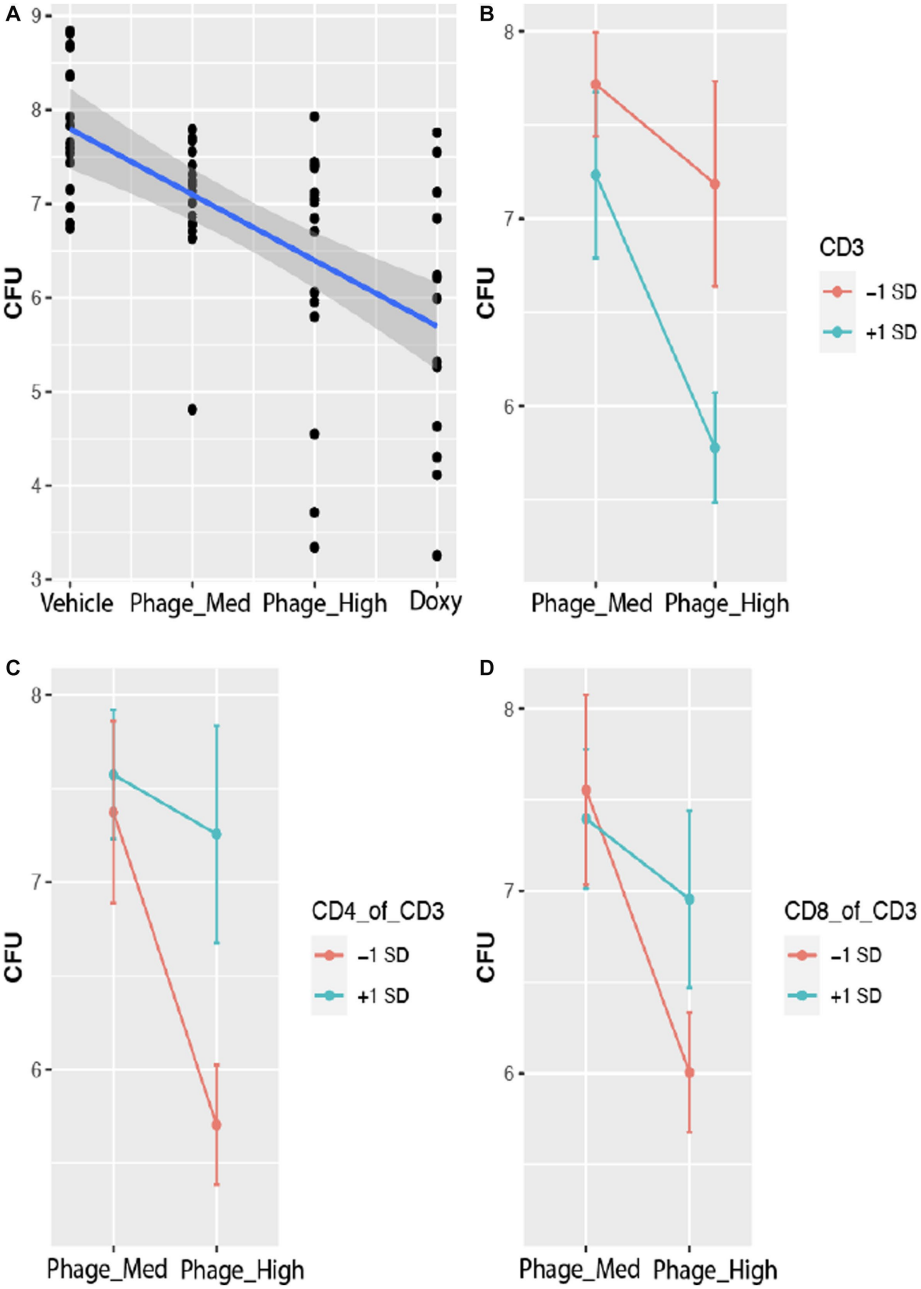
Host CD3^+^ T-cells modulate phage treatment effect on bacterial bioburden. Bacterial bioburden was measured based on the total colony forming units (CFU) of *A. baumannii* (strain AB5075:lux). CFU is log base 10 transformed data. **(A)** Effect plot of treatments on CFU. With the treatments varied from vehicle (control) to phage-Med, phage-HIGH, and as compared to doxycycline (Doxy), CFU decreased significantly in a linear fashion (*r* = −0.66; *p* = 0.005), indicating a signal of positive efficacy. **(B)** Effect plot of the treatment-CD3 interaction on CFU. Compared to phage-Med (and Vehicle), phage-High can significantly reduce CFU, when CD3 counts are one standard deviation above the mean of the CD3 counts. **(C,D)** The two interactions showed that phage-High can significantly reduce CFU, when the CD4:CD3 and CD8:CD3 ratios are one standard deviation below the respective means, compared to phage-Med (and Vehicle). Similar results were observed for Doxy.

To determine how multiple factors and their respective weighted values, such as treatment (TRT), time course (TIME), and host immune cell types, affect bacterial bioburden (CFU), we fit a linear mixed effects model. The results indicated that both TRT and TIME have significant effects on CFU (Table 1). Comparison of the treatments from Vehicle (control) to phage-Med, phage-High, and doxycycline (Doxy), CFU decreased significantly in a linear fashion (*r* = −0.66; *p* = 0.005), indicating a signal of positive efficacy (Figure 3A). The time course effect has a value of *p* (0.0002) that is 10-folder lower than that (*p* = 0.004) for the treatment effect, suggesting that TIME effect may capture, at least in part, the natural phase change of host immune responses and competency, in addition to the concomitant effect of the exogenous treatments (Table 1). Furthermore, when we examined the immunological profile of wounded and infected mice, we found that three treatment-immune cell (or ratio of cell types) interactions (TRT-CD3, TRT-CD4:CD3 ratio, and TRT-CD8:CD3 ratio) demonstrated significant (*p* < 0.05) effects on CFU (Table 1). The TRT-CD3 interaction indicated that compared to phage-Med (and Vehicle), phage-High can significantly reduce CFU, when CD3 is one standard deviation above the its overall mean (Figure 3B). Conversely, the other two interactions demonstrated that phage-High can significantly reduce CFU, when the CD4:CD3 and CD8:CD3 ratios are one standard deviation below their respective means (Figures 3C,D). Collectively, phage-High can significantly reduce bacterial bioburden, when there is a high percentage of CD3^+^ T-cells in the host immune cell population; otherwise, there is no significant effect. Therefore, the threshold of CD3^+^ T-cells present coupled with the dosage of phage treatment may induce a synergistic regimen that is critical to therapeutic efficacy, efficiency of bacterial clearance, and promotion of wound healing. Interestingly, the interactions between TIME and CD3 (or the ratios of cell types) are non-significant.

**Table 1:** A mixed effects model for bacterial bioburden.^1^

	Effect Est.	SE	*T*-value	d.f.	*p*-value
Intercept	29.598	16.214	1.825	63	0.073
TR	−17.340	5.733	−3.025	63	0.004**
TIME	−0.247	0.033	−7.441	63	0.000***
CD3	0.169	1.406	0.120	63	0.905
CD4:CD3	−7.839	7.342	−1.068	63	0.290
CD8:CD3	−4.155	2.992	−1.389	63	0.170
TRT|CD3	−1.302	0.582	−2.236	63	0.029*
TRT|CD4-of-CD3	8.184	2.817	2.905	63	0.005**
TRT|CD8-of-CD3	2.644	0.928	2.850	63	0.006**

^1^CD4:CD3 = CD4-of-CD3 ratio; CD8:CD3 = CD8-of-CD3 ratio.Significance levels are specified at 0.05 (*), 0.01 (**), and 0.001 (***) levels, respectively.

### 3.3. Dynamic association of bacterial bioburden and wound size is significantly modulated by the host immunological status

**Figure 4:**
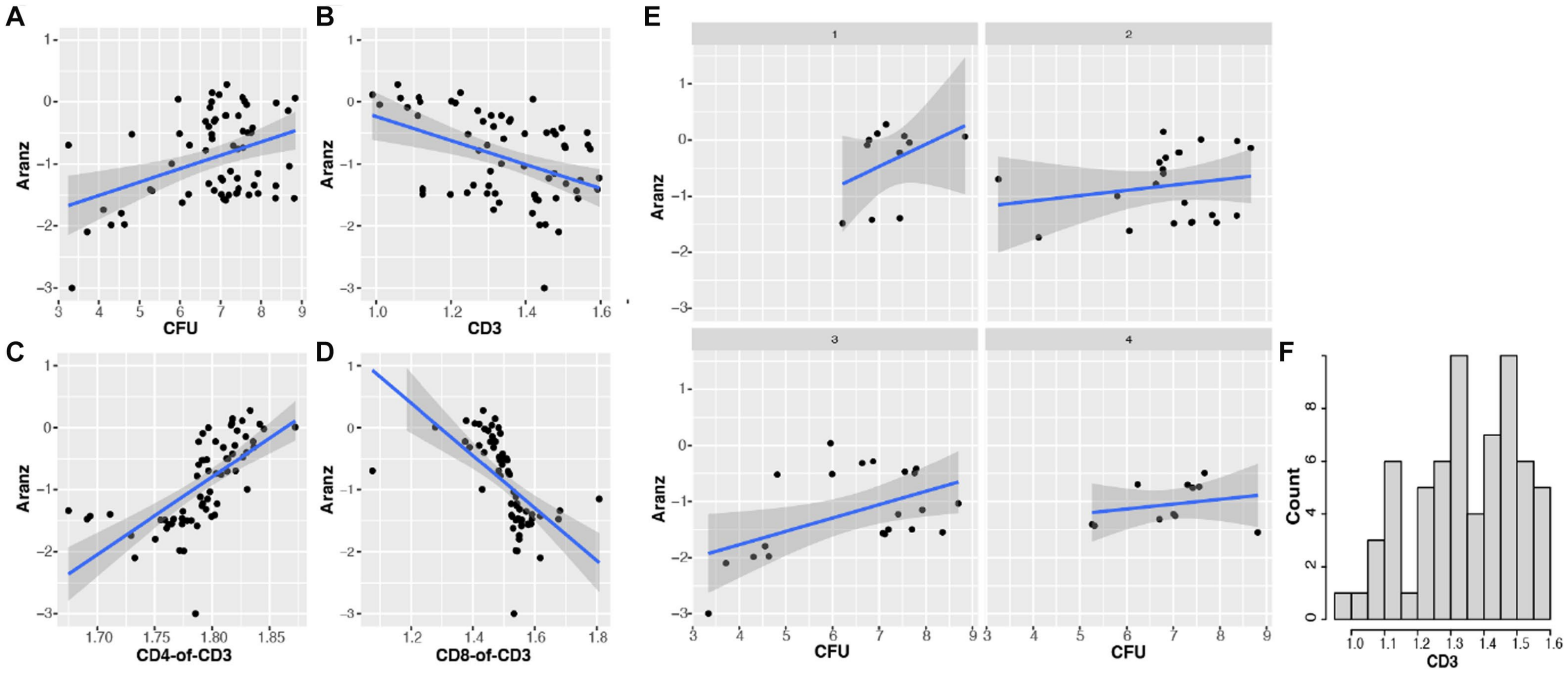
The association of bacterial bioburden (CFU) and wound size (Aranz) is modulated by host CD3^+^ T-cells. Data in this figure are log base 10 transformed. **(A)** The overall relationship between CFU and wound size is significantly positive (*r* = 0.396, *p* = 0.001). **(B)** The association of host CD3^+^ T-cells (or CD3) and wound size is significantly negative (*r* = −0.434; *p* = 0.0003). **(C,D)** The correlation between the CD4:CD3 ratio and wound size is positive (*r* = 0.666; *p* < 0.001), while that between the CD8:CD3 ratio and wound size is negative (*r* = −0.580, *p* < 0.001). **(E,F)** The distribution of log_10_ transformed CD3 (x) is shown in panel F. We then split the *x* variable into four groups: Group 1 (*x* < =1.15); Group 2 (1.15 < *x* < = 1.35); Group 3 (1.35 < *x* < = 1.5); and Group 4 (*x* > 1.5). The stratified CFU-wound size associations are presented in panel **(E)**. The regressions varied substantially in both slope and intercept. Corresponding correlation coefficients are 0.389 (*p* = 0.237), 0.194 (*p* = 0.287), 0.493 (*p* = 0.023), 0.238 (*p* = 0.481), respectively. Interestingly, only the correlation in group 3 is significant, indicating that a high percentage of host CD3^+^ T-cells in an appropriate range can drive the dynamic CFU-wound size association to the right direction of wounding healing.

**Figure 5:**
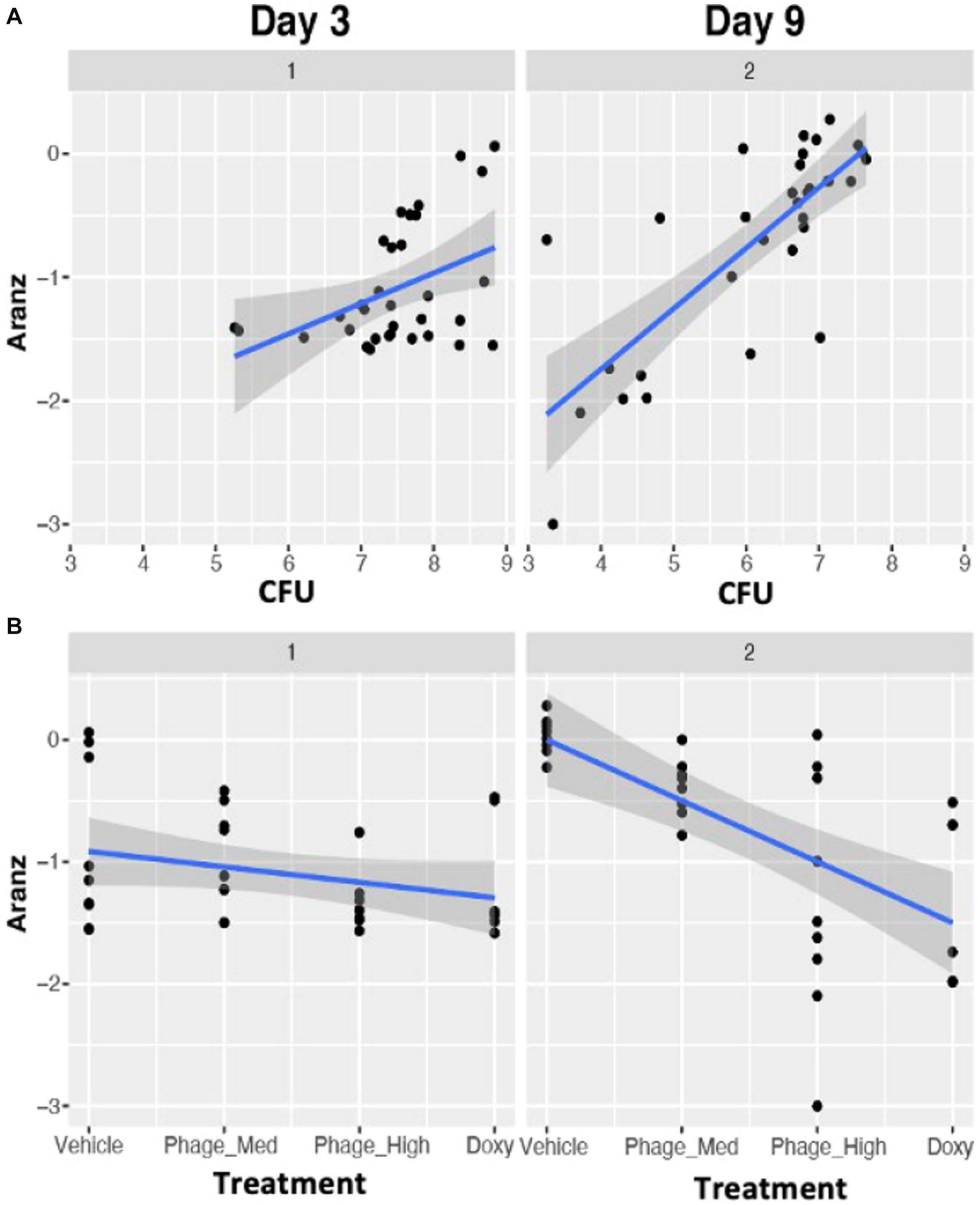
Time dependent associations between bacterial bioburden (CFU) and wound size **(A)**, and between treatments and wound size **(B)**. Compared to Day3, stronger such associations were observed on Day 9, indicating that in the first 3 days after the infection the host immune system has to handle the bacterial clearance, while on Day 9 the host is characteristic of the wound healing process. Data in this figure are log base 10 transformed.

Another objective of this study was to define and determine the dynamic association of bacterial bioburden and wound size, differentiated from confounding factors. Our results indicated that there is a significant positive correlation between CFU and wound size (r = 0.396, value of *p* = 0.001). This means that the more CFU present in a wound, the larger the wound size will become, or vice versa (Figure 4A). Furthermore, the regression of wound size variable on CFU is impacted by time course, with both intercept and slope significantly changed between the two time points, indicating a dynamic association of CFU and wound size (Figure 5A). To further investigate the underlying mechanisms of the CFU-wound size association, we examined the role of host CD3^+^ T-cells. In our assessment, we stratified the log_10_ transformed CD3 variable (*x*) into four groups: Group 1 (*x* < = 1.15); Group 2 (1.15 < x < = 1.35); Group 3 (1.35 < x < = 1.5); and Group 4 (*x* > 1.5) (Figure 4F). The CFU-wound size associations stratified by host CD3^+^ T-cells are shown in Figure 4E. The regressions differed substantially in both slope (0.40, 0.09, 0.24, and 0.09 for the four groups, respectively) and intercept (−3.24, −1.46, −2.72, and − 1.65, respectively). Interestingly, significant correlation (*r* = 0.493, *p* = 0.023) was observed for only Group 3 (Figure 4E), indicating that a higher percentage of host CD3^+^ T-cells in the appropriate range (1.35 < x < = 1.5) is essential for moving the dynamic association of bacterial bioburden and wound size to the right direction for decreasing CFU and promoting wound healing. Compared to the first group (*x* < = 1.15), however, the CFU-wound size association does not reach to the low end, indicating that a low level of CD3^+^ T-cells does not contribute to wound healing. Our results indicated that only when the host CD3^+^ T-cells (*x* on log10 scale) are in the appropriate range (1.35 < x < = 1.5), there is a strong correlation between CFU and wound size, indicating a hallmark of wound healing. Therefore, importance of the interaction between threshold of host CD3^+^ T-cells and phage dosage is further corroborated in bacterial clearance and wounding healing.

Furthermore, we observed a significant negative association between CD3 and wound size (*r* = −0.434; *p* = 0.0003) (Figure 4B). In an attempt to determine the impact of selected T-cell subsets, we found a positive association between CD4:CD3 ratio and wound size (*r* = 0.666; *p* < 0.001) (Figure 4C), while a negative correlation between CD8:CD3 ratio and wound size (*r* = 0.580, *p* < 0.001) (Figure 4D). Thus, our data indicates that CD8^+^ T-cells are the primary driving force of CD3^+^ T-cells responsible for reducing bacterial bioburden and minimizing wound size. These lines of evidence indicated that the dynamic association of bacterial bioburden and wound size is directly modulated by the host immunological status and selected immune populations.

### 3.4. Dynamic association of treatments and wound size is significantly modulated by the intensity of bacterial bioburden

The various treatments, which possessed different mechanisms of action in regards to antimicrobial activity, provided an important variable to examine the effects on wound size, with bacterial bioburden as a mediator. When the treatments varied from Vehicle to phage-Med, phage-HIGH, and as compared to doxycycline, wound size decreased significantly in a linear fashion (*r* = −0.493, value of *p* = 0.004), indicating a positive efficacy signal. However, the regression of wound size variable on treatments displayed different patterns between Day 3 and Day 9, with significant differences in both slope (−0.13 and −0.50 for Days 3 and 9, respectively) and intercept (−0.89 and 0.00, respectively) (Figure 5B). These clearly indicated a dynamic association of treatments and wound size. Compared to Day 3, we observed stronger association between treatment and wound size on Day 9, as is that between bacterial bioburden and wound size (Figure 5), indicating that in the first 3 days after the infection the host immune system has to deal with the bacterial clearance, while on Day 9 the host is characteristic of the wound healing process.

**Figure 6:**
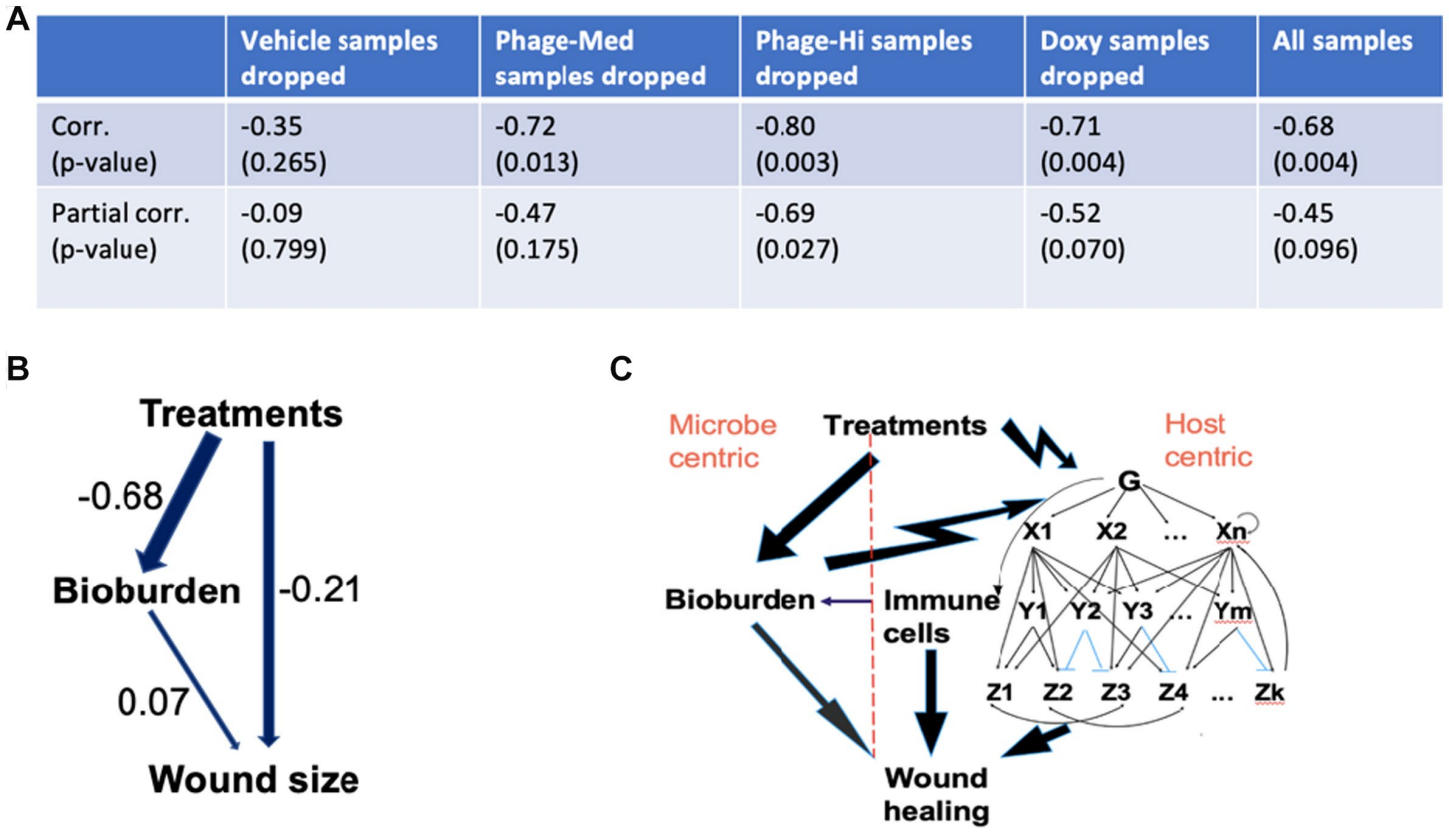
Mechanistic understanding of the wound healing system. **(A,B)** We scrutinized the treatments-wound size association by excluding one at a time the samples administered with a specific treatment. In each of the scenarios, we compared the treatments-wound size correlation to the partial correlation, given the status of bacterial bioburden (CFU). Regarding all of the samples, the correlation was significant (*p* = 0.004), the partial correlation had a value of *p* of 0.096, suggesting that there is still some effect from the right branch of the path model in panel **(B)**, although the value of *p* is >0.05. Therefore, the two-path model **(B)** can help explain the dynamic association of treatments and wound size that is modulated by the status of CFU. The numbers represent path coefficients or effect sizes. **(C)** Based on the interactions involved in the path model, we proposed an integrated systems biology model that has two branches: host- and microbe-centric. Regarding the host-centric branch, both cellular and molecular responses, especially in the immune system, play significant roles in coping with the bacterial bioburden and treatment stimuli. The arrows from treatments to bioburden and then to wound healing, and that from both cellular and molecular responses to wound healing represent only effects, as immune cells and both pro- and anti-inflammatory pathways can either promote or inhibit wound healing. In the gene regulatory subsystem, G represents sensors and global regulators, such as pattern recognition receptors that can identify molecules from pathogens (Takeuchi and Akira, 2010). *X* and *Y* are two different levels of transcription factors. Each *Z* is a large number of functionally related genes. In the subsystem, the top-down single-headed arrows denote activation, while the blue lines indicate suppression. In the gene regulatory subsystem, there are prevalent feed-forward loops, such as X1- > Y1- > Z1 and X1- > Z1, which play significant roles in shaping the responses to microbial infections. Double-headed arrows represent cross-talk between different biological pathways. The bottom-up single-headed and circular arrows indicate feedback regulation and autoregulation, respectively.

Since different treatments may contribute differentially in mean and variation to the overall treatment-wound size association (TWA), thus the TWA may be biased by a specific treatment, such as doxycycline. To avoid this, we then scrutinized the TWA by excluding one at a time the samples administered with a specific treatment (Figure 6A). In each of the scenarios, we compared the non-adjusted to the adjusted TWA, where the latter is the partial correlation given the status of CFU. Using this approach, we were able to estimate the relative contribution of CFU in mediation of the TWA. When the Vehicle (control) samples were dropped, both the correlation and partial correlation were non-significant, indicating that the control samples do not contribute to the TWA. When the phage-Med samples were removed, the correlation was significant (*p* = 0.013), but the partial correlation was non-significant (*p* = 0.175), implying that CFU as a mediator can explain away the TWA. In contrast, when the phage-High samples were taken away, both the non-adjusted and adjusted correlations were significant (*p* = 0.003 and 0.027, respectively), demonstrating that CFU as a mediator cannot explain away the TWA. Therefore, between the two phage dosages (phage-Med and phage-High), there existed a significant difference in path models: a model with only the left branch in Figure 6B against another model with both the left and right branches. Regarding all of the samples, the correlation was significant (*p* = 0.004), the partial correlation had a value of *p* = 0.096, suggesting that there is still some effect from the right branch, although the value of *p* is >0.05. Therefore, a path model is derived to explain the dynamic association of treatments and wound size that is modulated by the status of CFU (Figure 6B). This model intrigued us to develop a systems biology for wound healing.

## 4. Discussion

Using an established mouse wound infection model and multiple treatment modalities, we uncovered that the interplay between bacterial burden (CFU) and host immune responses is bidirectional. Similar to previous studies that the presence of bacterial bioburden can stimulate the host to illicit cellular and molecular responses from various cell types (Grice and Segre, 2012; Desgeorges et al., 2019), our results indicate the host responses to CFU have a dynamic phase change from pro-inflammation to anti-inflammation (Figure 2). On the other hand, phage treatment delivered at 5 × 10^8^ total PFU (phage-High) can significantly reduce CFU, compared to phage-Med (5 × 10^7^ total PFU), when there is a high percentage of CD3^+^ T-cells in the host immune cell population. Furthermore, we revealed that when the T-cells (x on the log10 scale) are in the appropriate range (1.35 < x < = 1.5), there is a strong correlation between CFU and wound size (Figure 4E), indicating a signal for wound healing. These new findings clearly signify the importance of CD3^+^ T-cells – phage dosage synergy in the wound healing system, thus laying the foundation for future exploration of phage treatment in wound healing of human patients.

Wound healing is impacted by a complex system involving treatments, microbial bioburden, and competency of the host immune system (or “host-microbe-treatment” triangle). Furthermore, time series after the injury provides another variable within the multifaceted system that governs the outcomes of successful closure or dehiscence (failure) of wounds. For example, compared to Day 3, we observed stronger associations between bacterial bioburden and wound size, and between treatment and wound size (Figure 5), indicating that in the first 3 days after the infection the host immune system has to handle the stress of bacterial clearance, while on Day 9 the host is characteristic of the wound healing process.

We used both reductionist and holistic approaches to exploring and understanding the wound healing system. Since little is known in the research community about the dynamic interactions between the key components involved in the wound healing system, thus we took a first step to explore the interplay between: (1) bacterial bioburden and host immune responses, (2) bacterial bioburden and wound size, and (3) treatments and wound size. The pairwise interactions are not only time dependent as described above, but modulated by the other component(s) involved in the system. Therefore, we had to use sophisticated statistical methods, such as mixed effects linear regression modeling, covariate stratification, and path analysis, to handle the cofactors. After the reductionist analysis, we then initiated to explore and represent the full picture about the wound healing system.

The dynamic interactions, especially the bi-branch path model (Figure 6B), provoked us to develop a systems biology model for wound healing. The right branch in the path model (Figure 6B) is not a direct path biologically, as multiple layers of variables are absent from the present study, such as gene regulation and mRNA expression. Our study indicated that host immune cell types, such as both relative and absolute CD3^+^ T-cells are among the key players involved in the wound healing system. In a collaborative research project between the first author and Dr. Rachel Sparks at the National Institute of Allergy and Infectious Diseases (NIAID), we identified that different human immune cell types (such as CD4 and CD8) are characteristic of specific signatures of transcription factor activities that orchestrally regulate pro- and anti-inflammatory biological pathways (data not shown). We then suspect that gene regulation and expression in specific immune cell types, such as CD3^+^ T-cells, play significant roles in the wound healing system in humans and mice. Similarly, human regulatory T-cells (Tregs) are crucial coordinators of the immune responses to injury in several organs (Boothby et al., 2020). Above all, the host needs to be able to censor the outside stress signals, such as bacterial bioburden and treatment challenging, followed by triggering inducible immune responses at both cellular and molecular levels (Takeuchi and Akira, 2010; Iwasaki and Medzhitov, 2015; Paludan et al., 2021). Our results indicated that there exists a dynamic phase change of the host immune responses to the infections (Figure 2). The dynamic phase change in cytokines and chemokines along the downstream biological pathways results from upstream gene regulation mechanisms, such as the prevalent feed-forward loops in the gene regulatory system (Mangan et al., 2006; Li et al., 2015). The feed-forward loops facilitate fast responses to outside stimuli (Mangan et al., 2006). Therefore, we proposed an integrated systems biology model for wound healing (Figure 6C), with both host- and microbe-centric branches corresponding to the path model (Figure 6B). This model is coupled and evolved with the phase changes of immune cell types, such as macrophages at an early stage and T-cells at a later stage. It is noteworthy that the dynamic association of bacterial bioburden and wound size is too complicated to be captured by any single protein biomarker. However, it can be represented by the phase change of the host immune responses, where cytokine and chemokine biomarkers are coupled with corresponding immune cell types. When there is a change from the proinflammation-enriched phase 1 to the anti-inflammatory phase 2, we observed decreasing CFU and wound size, indicating a positive signal of wound healing.

As antibiotic resistant strains of bacteria become more prevalent, the development of alternative sustainable treatments, such as bacteriophage (phage), is key to augmenting antimicrobial drugs. Compared to broad-spectrum antibiotics, phage therapy has the advantage of being highly specific toward target bacterial pathogens without adversely affecting the host or host commensal microbiota (Sarker et al., 2017). A recent study on various murine models, such as healthy immunocompetence, MyD88-deficiency, lymphocyte-deficiency, and neutrophil-depletion, indicated that neutrophil-phage synergy is essential for infection clearance and the resolution of pneumonia (Roach et al., 2017). This new “immunophage synergy” concept provides clear evidence of microbe-host immune system interaction (Roach et al., 2017). In the present study, we identified that phage treatment delivered at 5 × 10^8^ total PFU can significantly reduce bacterial bioburden, when there is a high percentage of CD3^+^ T-cells in the immune cell population of mice; otherwise, there is no such effect. This CD3^+^ T-cells-phage dosage synergy plays an important role in reducing bacterial burden and increasing wound healing, thus laying the foundation for future exploration in using phage treatments in wound healing. This synergy may be attributed to the dynamic interactions involved in the wound healing systems, especially the associations between treatments and host immune responses, and between bacterial bioburden and the immune responses. Interestingly, our modeling results showed that the neutrophil (or Ly6G) cell type had non-significant effects on bacterial bioburden (data not shown). This may be explained by the anti-Ly6G administered 2 days before the experiments (Figure 1). Further studies are needed to reveal the mechanisms underlying the dosage effect. Additional experiments are also needed to decipher the contribution of CD3^+^ T-cells in treatment efficacy and clearance of infection, examine other immune cell types, such as monocytes and neutrophils, and investigate the effectiveness of combination treatments (phage + antibiotics) on wound healing at lower doses of phage.

The dynamic bacteria-bacteriophage interactions were not explored in the present study. Investigation of such interactions will contribute to the understanding of how the wound etiology and microbiome as a whole is related to host immune responses in wound healing (Verbanic et al., 2022; Marchi et al., 2023). For successful therapy, it is critical to understand how bacterial pathogens might become resistant to phages and, therefore, recalcitrant to treatment.

There is a limitation in the present study. The power of this study to explore the wound healing system was limited by only one inbred mouse strain used. Different genetic backgrounds may play varied roles in shaping the dynamic interactions within the wounding healing system. More studies are needed by using heterogeneous animal model cohorts, such as the Collaborative Cross mice (Threadgill et al., 2011). The heterogeneous genetic background can mimic the human patient population and reproduce the complexity of human wounds (Threadgill et al., 2011). Furthermore, big data from multi-omics are needed to better understand the wound healing system. Collectively, However, our findings show strong relationships between host immune competency, therapeutic efficacy, bacterial clearance, and wound healing.

## Data availability statement

The raw data will be made available by all reasonable requests and in accordance with DoD guidelines for data sharing.

## Ethics statement

The animal study was approved by the experiments were conducted according to the compliance policy set by the Committee of Laboratory Animal Resources and Institutional Animal Care and Use Committee (IACUC) at the Naval Medical Research Center. The study was conducted in accordance with the local legislation and institutional requirements.

## Author contributions

MR and MS designed the experiments and contributed to manuscript writing. SS contributed to the experimental design. MR conducted the experiments. RL carried out the data analysis, proposed the integrated system biology model, and wrote the manuscript. SG contributed to data analysis and management. KM and BP contributed to data analysis or literature search. All authors contributed to the article and approved the submitted version.

## Funding

The research project was supported by a grant (W0302_19_NM) from the Military Infectious Diseases Research Program (MIDRP): Development of machine learning algorithms in animal models for prediction of wound outcomes.

## Conflict of interest

The authors declare that the research was conducted in the absence of any commercial or financial relationships that could be construed as a potential conflict of interest.

## Publisher’s note

All claims expressed in this article are solely those of the authors and do not necessarily represent those of their affiliated organizations, or those of the publisher, the editors and the reviewers. Any product that may be evaluated in this article, or claim that may be made by its manufacturer, is not guaranteed or endorsed by the publisher.

## Author disclaimer

The contents of this manuscript are the sole responsibility of the authors and do not necessarily reflect the views, opinions or policies of Uniformed Services University of the Health Sciences (USUHS), The Henry M. Jackson Foundation for the Advancement of Military Medicine, Inc., the Department of Defense (DoD) or the Departments of the Army, Navy, or Air Force. Mention of trade names, commercial products, or organizations does not imply endorsement by the U.S. Government. The authors declare no competing interests. Some of the authors of this work are military service members or employees of the U.S. Government. This work was prepared as part of their official duties.
